# Betaine in ameliorating alcohol-induced hepatic steatosis

**DOI:** 10.1007/s00394-021-02738-2

**Published:** 2021-11-24

**Authors:** Aisha Rehman, Kosha J. Mehta

**Affiliations:** 1 Faculty of Life Sciences and Medicine, King’s College London, London, UK; 2 Centre for Education, Faculty of Life Sciences and Medicine, King’s College London, London, UK

**Keywords:** Betaine, Alcohol, Alcoholic liver disease, Alcohol-associated liver disease, AALD, Steatosis, Fatty liver

## Abstract

Alcohol-associated liver disease (AALD) is one of most common chronic liver diseases. Hepatic steatosis is the earliest stage in AALD pathological spectrum, reversible by alcohol abstinence. Untreated steatosis can progress to steatohepatitis, fibrosis and/or cirrhosis. Considering the difficulties in achieving complete abstinence, challenges in disease reversal at advanced stages, high costs of AALD management and lack of standardised prescribed medications for treatment, it is essential to explore low-cost natural compounds that can target AALD at an early stage and halt or decelerate disease progression. Betaine is a non-hazardous naturally occurring nutrient. Here, we address the mechanisms of alcohol-induced hepatic steatosis, the role of betaine in reversing the effects i.e., its action against hepatic steatosis in animal models and humans, and the associated cellular and molecular processes. Accordingly, the review discusses how betaine restores the alcohol-induced reduction in methylation potential by elevating the levels of S-adenosylmethionine and methionine. It details how betaine reinstates alcohol-induced alterations in the expressions and/or activities of protein phosphtase-2A, FOXO1, PPAR-α, AMPK, SREBP-1c, fatty acid synthase, diacylglycerol transferase-2, adiponectin and nitric oxide. Interrelationships between these factors in preventing de novo lipogenesis, reducing hepatic uptake of adipose-tissue-derived free fatty acids, promoting VLDL synthesis and secretion, and restoring β-oxidation of fatty acids to attenuate hepatic triglyceride accumulation are elaborated. Despite its therapeutic potential, very few clinical trials have examined betaine’s effect on alcohol-induced hepatic lipid accumulation. This review will provide further confidence to conduct randomised control trials to enable maximum utilisation of betaine’s remedial properties to treat alcohol-induced hepatic steatosis.

## Introduction

With approximately two billion individuals drinking alcoholic beverages worldwide, alcohol-associated liver disease (AALD) is one of the most common chronic liver diseases. It is a result of prolonged excessive alcohol consumption and one of the leading reasons for liver transplantation in the US; liver being the most affected organ as it is the main site of ethanol metabolism. Like other chronic liver diseases, AALD pathological trajectory includes the overlapping and progressive stages of hepatic steatosis, steatohepatitis, fibrosis, and/or cirrhosis with associated complications like ascites and/or variceal bleeding and encephalopathy. Untreated cases could progress to liver failure or hepatocellular carcinoma [1]. Alcoholic hepatitis is associated with high mortality and healthcare costs that run in billions [2]. Alongside, more than 50% cirrhosis-related deaths are attributed to alcohol consumption [3, 4]. To reduce the costs and mortality associated with the advanced stages, it is necessary to halt disease progression at an early stage.

Alcohol-induced hepatic steatosis is one of earliest manifestations of acute or chronic alcohol consumption [5] and therefore makes an excellent target for therapeutic intervention. It is characterised by accumulation of excess fat in the liver, i.e., deposition of lipid droplets consisting of triglycerides and cholesterol esters [6]. Steatosis (and mild alcoholic hepatitis) can be reversed by alcohol abstinence but this stage is usually asymptomatic [5], thereby increasing the probability of under-diagnosis. Untreated steatosis can sensitise the liver to progressive injuries [7] and at the advanced stages, disease reversal/regression becomes extremely difficult.

As such, there are several challenges at the therapeutic front, one being the absence of a standardized prescribed medication for AALD or its associated complications. Hitherto, AALD management has focused mainly on alcohol abstinence, which can halt disease progression [5], but complete abstinence can be difficult to achieve. Certain drugs can reduce alcohol craving, others such as Nalmefene can manage alcohol dependence and reduce alcohol consumption [8] but their effects may differ. Disulfiram can prevent relapses, but it can cause liver damage. There are other drugs, but most have not been studied in patients with alcoholic hepatitis and cirrhosis. Nutritional therapy (e.g. selenium, beta carotene, vitamins A, C, E, B6 and B12) has not shown great benefits [5, 9]. Corticosteroid treatment has been used for moderate to severe alcoholic hepatitis but results have been variable [10]. Liver transplantation and resection remain the ultimate treatment options for any end-stage liver disease, but these procedures are prone to complications, as expected.

Collectively, this urges to explore non-toxic low-cost natural therapeutic compounds that target the early stage of steatosis and are safe to use as an adjunct to the current AALD treatment/management strategies so that disease progression can be halted, decelerated and/or reversed.

Betaine is a natural nutrient which has shown protective effects against hepatic fat deposition. Accordingly, this review focuses on the beneficial effects of betaine and the associated mechanisms underlining betaine-mediated amelioration of alcohol-induced hepatic steatosis.

## Contextual therapeutic potential of betaine

Betaine is found in various food sources, such as sugar beet, bread, spinach, wheat, and shellfish. It is low-cost, stable, non-toxic, shows high efficacy and has been approved for human consumption. While betaine can be obtained from dietary sources, it can also be synthesized endogenously by the kidneys and liver [11]; the latter shows most abundance of betaine and its transporter. It is maintained in the circulation at a median concentration of 27.8 µmol/L and acts as an osmolyte [6, 11, 12].

In line with its myriads of functions in human physiology, betaine resolved alcohol-induced hepatitis and steatosis after liver transplantation in a 40-year-old woman [13]. Also, when betaine was administrated in partially hepatectomized rats, it promoted liver regeneration whereby the liver regained its original weight in 7 days after surgery [14]. Alongside, it has exhibited great therapeutic potential in clinical trials for non-alcoholic steatohepatitis [15] and homocystinuria [16]. Generally, betaine has shown hepatoprotective properties against liver injury by enhancing antioxidant mechanisms and adipose tissue function, while reducing oxidative stress, endoplasmic stress, liver fibrosis and necrosis [17]. It has also shown to attenuate alcohol-induced pancreatic steatosis and high-fat-diet-induced hepatic steatosis in animals [18, 19].

AALD progression can be halted, and disease progression reversed upon cessation of alcohol consumption. However, those who chronically consume alcohol often find it difficult to abstain completely. Therefore, more lenient approaches are being considered. For example, recently, comparison of abstinence-based and non-abstinent treatment approaches did not support abstinence as the sole approach. Instead, it proposed controlled drinking supported by psychotherapy as an additional AALD management approach [8]. Betaine can be useful in such cases of partial abstinence because experiments in animal models have shown that betaine supplementation alongside ethanol-feeding can still decrease alcohol-induced hepatic triglyceride levels/accumulation, prevent and partially reverse alcoholic fatty liver [20], and generally improve liver health [7, 21–23]. Mechanistically, betaine improves hepatic lipid metabolism by stimulating fatty acid oxidation and lipid secretion whilst inhibiting fatty acid synthesis in the liver [24].

## Mechanisms underlying alcohol-induced steatosis and betaine-mediated mending

Alcohol-induced hepatic steatosis, i.e. hepatic fat accumulation is caused by four main events as listed below [6]:

i. Increased breakdown of adipose tissue into free fatty acids that are subsequently taken up by the liver and converted to triglycerides [10].
ii. Increased de novo hepatic lipogenesis.
iii. Decreased mitochondrial fatty acid oxidation [25].
iv. Decreased synthesis and secretion of very-low density lipoprotein (VLDL).

**Fig. 1:**
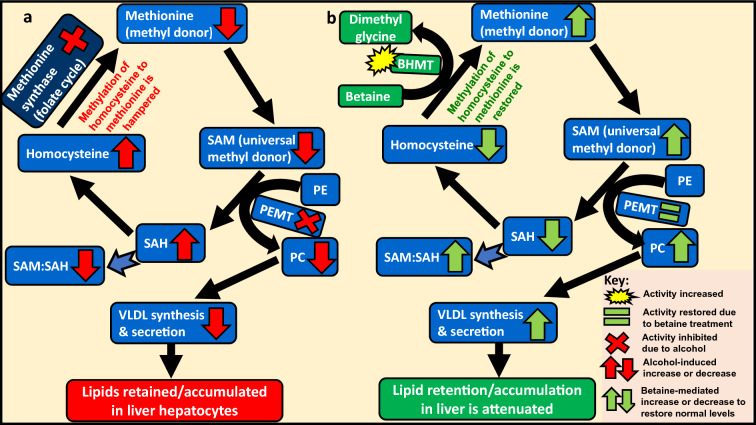
Alcohol-induced impairment of methionine cycle and betaine-mediated repair. Methylation is essential for normal body functionality and homeostasis. Methionine metabolism/cycle is central to this process as it generates methyl-group donors. **a** Alcohol hampers methionine cycle by increasing or decreasing levels/activities of various components of this cycle, eventually leading to hepatic lipid accumulation. **b** Betaine repairs the alcohol-induced alterations and thereby attenuates lipid accumulation in the liver. *BHMT* betaine-homocysteine methyltransferase, *PC* phosphatidylcholine, *PE* Phosphatidylethanolamine N-methyltransferase, *PEMT* Phosphatidylethanolamine N-methyltransferase, *SAH* S-adenosylhomocysteine, *SAM* S-adenosylmethionine, *VLDL* very-low density lipoprotein

Betaine can amend all four mechanisms and reduce hepatic triglyceride accumulation. A characteristic feature of betaine is its ability to act a methyl-group donor, particularly relevant and operational in the liver. This ability allows betaine to participate in the methionine cycle. Betaine’s lipotropic characteristic (i.e. ability to prevent hepatic lipid accumulation) is largely attributed to its ability of restoring methionine homeostasis and repairing the alcohol-induced reduction of methylation potential (Fig. 1) [11].

## Betaine restores alcohol-induced decrement in methylation potential

Methionine is a methyl-group donor and an essential amino acid. Its metabolism/cycle generates the universal methyl-group donor S-adenosylmethionine (SAM), which is also a precursor of the antioxidant glutathione. Subsequent metabolite S-adenosylhomocysteine (SAH) is converted to homocysteine, which is toxic and needs to be removed from plasma. This is achieved by transferring methyl groups to homocysteine (either via methionine synthase of the folate cycle or endogenous cytoplasmic betaine) to regenerate methionine. In turn, this produces more SAM and eliminates homocysteine. As the cycle continues, SAM:SAH ratio is maintained at high levels and homocysteine levels are regulated (kept low) via its conversion to methionine (Fig. 1) [11].

Alcohol consumption reduces the levels of endogenous hepatic betaine and SAM, increases levels of SAH and plasma homocysteine, and inhibits methionine synthase activity. SAM:SAH ratio is reduced (Fig. 1a) [11, 22, 26]. Supplemented betaine re-methylates homocysteine to form methionine and dimethyl glycine via increased activity and upregulation of the enzyme betaine-homocysteine methyltransferase (BHMT) [11, 22, 27]. This not only replenishes methionine levels but also generates more SAM and reduces/regulates SAH levels. Thus, SAM:SAH ratio is increased and restored, and levels of plasma homocysteine are reduced and maintained (Fig. 1b) [9, 20, 22, 28, 29]. Also, BHMT levels and activity affect the methylation status. In mice, decrease in BMHT hampered the removal of methyl groups from betaine and this was associated with increased fatty liver and hepatocellular carcinoma [11, 26].

**Fig. 2:**
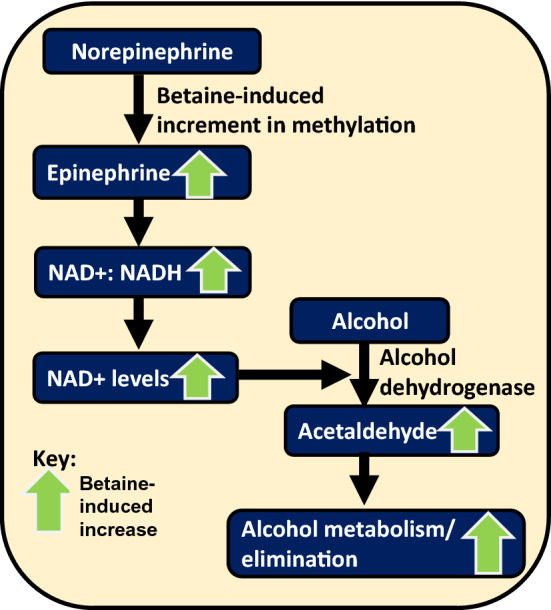
Betaine-mediated methylation of epinephrine. Betaine-mediated methylation of norepinephrine to epinephrine increases the levels of NAD +, which can fuel the catalytic conversion of alcohol to acetaldehyde by alcohol dehydrogenase. Thus, betaine can aid in eliminating alcohol by providing methyl groups to norepinephrine

Alcohol-induced reduction in SAM:SAH ratio reduces the overall methylation potential and unfavourably affects methylation of many important hepatic factors. For example, the conversion of norepinephrine to epinephrine, which is a methylation event. Betaine increases the methylation of norepinephrine leading to increased production of NAD +, which further helps alcohol dehydrogenase to metabolise alcohol into acetaldehyde and eventually promote its elimination (Fig. 2) [30]. Other examples of alcohol-induced hypo-methylation in facilitating hepatic lipid accrual and betaine-mediated restoration of methylation potential have been cited in the subsequent sections.

**Fig. 3:**
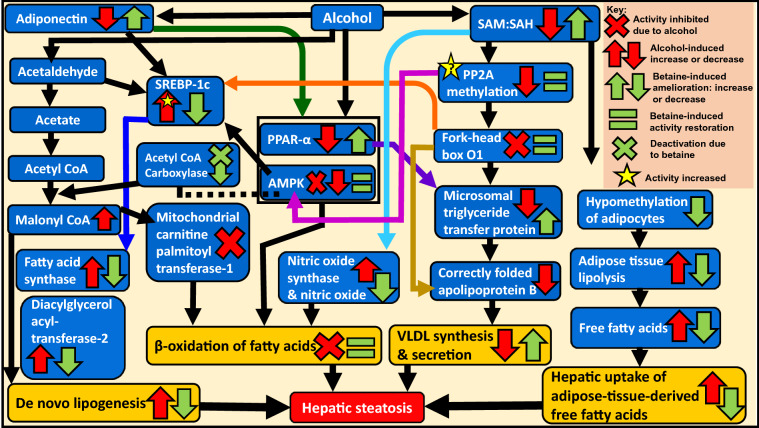
Core events and factors of alcohol-induced hepatic steatosis and betaine-mediated amelioration. Key interrelated events and factors that cause alcohol-induced excess hepatic triglyceride accumulation are depicted. Alcohol-induced events include (i) increased hepatic de novo lipogenesis, (ii) decreased/hindered mitochondrial fatty acid oxidation, (iii) reduced synthesis and secretion of VLDL, and (iv) increased hepatic uptake of adipose-tissue-derived free fatty acids. There are several mechanisms and factors that facilitate these events. This figure also shows selected mechanisms of betaine-mediated amendments to these alcohol-induced effects. Interestingly, alcohol metabolism generates excessive amount of malonyl-CoA. This inhibits mitochondrial carnitine palmitoyl transferase-1 [25], the enzyme essential for β-oxidation of fatty acids, thereby contributing to alcohol-induced impairment in fatty acid oxidation. Dotted line indicates the relation between AMPK and acetyl-CoA carboxylase; activation of AMPK deactivates (phosphorylates) acetyl-CoA carboxylase and thereby partly inhibits hepatic fatty acid synthesis [34]. Yellow star with question reflects the apparent contradiction between alcohol-induced increment in activity of PP2A and alcohol-induced decrease in methylation of PP2A, which needs further investigation. *AMPK* AMP-activated protein kinase, *PP2A* Protein phosphatase-2A, *PPAR* Peroxisome proliferator activated receptor, *SAH* S-adenosylhomocysteine, *SAM* S-adenosylmethionine, *SREBP* Sterol regulatory element binding protein, *VLDL* Very-low density lipoprotein

There are numerous signalling events, enzymes, and transcription factors that mediate alcohol-induced triglyceride and cholesterol accumulation in the liver (Fig. 3). Subsequent sections focus on selected mechanisms and factors that have shown betaine-mediated amendments in the context of alcohol-induced hepatic steatosis.

## Betaine reduces alcohol-induced adipose tissue lipolysis

Adipose tissue is an important energy reserve. Chronic alcohol consumption reduces adipose tissue mass by increasing fat breakdown (lipolysis) [10]. This generates excess free fatty acids which are taken up by the liver, thereby accelerating steatosis and AALD pathogenesis (Fig. 3). Hypo-methylation in adipocytes/adipose tissue contributes to increased adipose tissue lipolysis. In animal models of AALD, betaine has shown to exert hepatoprotective effects by improving the methylation status of adipose tissue and its function, which in turn reduces the alcohol-induced increment in adipocyte lipolysis (Fig. 3) [31].

## Betaine attenuates alcohol-induced elevation in de novo hepatic lipogenesis

Hepatic lipogenesis utilises sterol regulatory element binding protein (SREBP)-1. This lipogenic transcription factor facilitates de novo lipogenesis in the liver and other tissues. It regulates lipid synthesis and increases hepatic expression of lipogenic enzymes like fatty acid synthase, stearoyl CoA desaturase, acetyl-CoA carboxylase [32]. Acetaldehyde, an intermediate metabolite of alcohol metabolism activates and increases the expression of SREBP-1c (Fig. 3) [7, 25]. No wonder, SREBP-1 has been implicated in the development of fatty liver in alcoholic and non-alcoholic steatohepatitis [33]. This alcohol-induced increment in SREBP-1c expression is downregulated by betaine (Fig. 3) [7], thereby reducing de novo hepatic lipogenesis and preventing hepatic fat accumulation.

Alcohol is metabolised to acetyl-CoA, which participates in de novo lipogenesis (Fig. 3). Essentially, fatty acid synthase aids in fatty acids synthesis from acetyl-CoA and palmitate. Alcohol increases the expression of fatty acid synthase whereas betaine supresses this increment in the liver (Fig. 3). Another enzyme involved in fatty acid synthesis is diacylglycerol acyltransferase-2. It is a rate-limiting enzyme that catalyses the last step in triglyceride synthesis whereby diacylglycerol is esterified with a fatty acid. Alcohol increases the expression of diacylglycerol acyltransferase to promote triglyceride synthesis, but betaine supresses its expression in the liver (Fig. 3) [7] and thus helps evade liver lipid synthesis. Also, acetyl-CoA carboxylase, which catalyses the conversion of acetyl-CoA to malonyl-CoA is the rate-limiting enzyme in hepatic de novo lipogenesis. In animal models, betaine has shown to attenuate high-fat-sucrose-induced increase in hepatic acetyl-CoA carboxylase and inhibit its activity [34], eventually reducing hepatic lipogenesis. A similar mechanism of action of betaine could be envisaged in case of an alcohol-feed (Fig. 3).

## Betaine repairs alcohol-induced impairment in fatty acid oxidation and uptake

Peroxisome proliferator activated receptor (PPAR)-α is a transcription factor that senses lipids in the liver. It transcribes several genes involved with β-oxidation of fatty acids, fatty acid uptake and transport, and lipoprotein production [35]. It is implicated in the development of fatty liver in alcoholic and non-alcoholic steatohepatitis [33]. Alcohol decreases [25]**,** but betaine increases and restores PPAR-α expression by decreasing methylation of its promoter (Fig. 3) [36]. Subsequently, this would increase the mRNA expression of its target genes that help implement its aforementioned functions.

AMP-activated protein kinase (AMPK) regulates lipid metabolism [11]. Its activation (phosphorylation) decreases lipogenic enzymes and lipogenic transcription factors such as SREBP1, inhibits hepatic fatty acid synthesis partly via inhibition of acetyl-CoA carboxylase [37], increases β-oxidation of fatty acids in the liver mitochondria, and generally improves mitochondrial function that helps prevent and ameliorate hepatic steatosis [38] (Fig. 3). Alcohol reduces AMPK expression [39] and inhibits its activity [40]. Betaine increases AMPK phosphorylation (activation) and thereby restores AMPK activity (Fig. 3) [34]. As expected, this restoration activates genes involved in fatty acid transport and oxidation, and decreases fatty acid synthesis. Activated AMPK targets acetyl-CoA carboxylase, which leads to the latter’s phosphorylation (deactivation). Also, betaine has shown to reduce acetyl-CoA carboxylase levels in the liver. Thus, increased betaine-induced hepatic activation of AMPK is associated with attenuation of hepatic steatosis in animals (Fig. 3) [11, 34].

Adiponectin maintains lipid homeostasis, inhibits lipid synthesis, and stimulates fatty acid oxidation, partly by activating AMPK and PPAR-α, and by inhibiting SREBP-1. Alcohol decreases adiponectin secretion and inhibits hepatic expression of adiponectin receptors; particularly adiponectin-receptor-1. Betaine restores serum adiponectin levels and partially restores levels of adiponectin-receptor-1 [7, 41]. This eventually aids in restoring β-oxidation of fatty acids and attenuating de novo lipogenesis (Fig. 3).

In AALD, hepatic mitochondria alter in function and structure. For example, alcohol-affected mitochondria are swollen [7], elongated, show increased mitochondrial respiration and decreased levels of mitochondrial proteins involved in oxidative phosphorylation. Betaine can re-establish the original functions/mitochondrial activity, and thus prevent alcohol-induced mitochondrial injury [7, 11, 24]. Thus, betaine protects hepatocyte mitochondria in animal models of acute and chronic liver injury [17].

An enzyme-molecule pair that affects mitochondrial health in AALD is nitric oxide synthase and nitric oxide, a signalling molecule. Probably, alcohol-induced decrement in SAM:SAH ratio induces the generation of both in the liver. This not only inhibits aldehyde dehydrogenase-2 and hampers alcohol metabolism but also inhibits mitochondrial enzymes like 3-ketoacyl-CoA thiolase, a facilitator of mitochondrial β-oxidation of fatty acids [42]. Betaine blocks the alcohol-induced generation of these moieties and thereby helps preserve mitochondrial function and promote β-oxidation of fatty acids (Fig. 3) [23].

## Betaine restores alcohol-induced reduction in VLDL synthesis and secretion

Hepatic lipid accumulation involves impaired VLDL export. VLDL transport is the means of transporting triglycerides and cholesterol from the liver/hepatocytes to the circulation, a process that removes/reduces intrahepatic lipid levels. Alcohol-induced reduction in SAM:SAH ratio unfavourably affects phosphatidylethanolamine N-methyltransferase (PEMT)-mediated methylations of phosphatidylethanolamine N-methyltransferase (PE) to form phosphatidylcholine (PC). PC is an essential component of VLDL. Alcohol reduces PEMT activity, which reduces PC levels and thereby reduces VLDL synthesis and secretion. This results in impaired transport of triacylglycerols from the liver into the circulation and promotion of alcohol-induced hepatic steatosis (Fig. 1a). Betaine restores PEMT activity, PC levels, and consequently, VLDL synthesis and secretion (Fig. 1b) [6, 20, 28, 43].

Apolipoprotein-B serves to assemble lipids using microsomal triacylglycerol transfer protein to form hepatic triacylglycerol that can be secreted as VLDL. Thus, VLDL synthesis and secretion is additionally regulated by levels of apolipoprotein-B and the activity of microsomal triglyceride transfer protein. Alcohol reduces the activity of hepatic PPAR-α [41] leading to downregulation of microsomal triglyceride transfer protein [35] causing subsequent reduction in VLDL synthesis and secretion (Fig. 3). While betaine can elevate PPAR-α expression [36] to restore microsomal triglyceride transfer protein, in rats (Fig. 3), dietary betaine increased the expressions of hepatic BHMT (Fig. 1b) and apolipoprotein-B, elevated VLDL secretion and thereby reduced hepatic triacylglycerol (Fig. 3) [44].

Yet another example of the undesirable effect of alcohol-induced hepatic hypo-methylation involves protein phosphatase-2A and the activity of Forkhead box O1 (FOXO1), a transcription factor that contributes to hepatic lipid metabolism. Alcohol-induced hypo-methylation reduces the methylation of protein phosphatase-2A catalytic C subunit, which increases phosphorylation (inactivation) of FOXO1 (Fig. 3) [45]. FOXO1 de-phosphorylation (activation) allows its translocation into the nucleus [46], while phosphorylated inactive FOXO1 is excluded from the nucleus. Activated FOXO1 can transcriptionally activate microsomal triglyceride transfer protein whereas inactivated FOXO1 reduces microsomal triglyceride transfer protein and has shown to affect apolipoprotein-B levels in vitro (Fig. 3). Thereby, inactivated FOXO1 (a downstream result of alcohol exposure) restricts/reduces VLDL assembly (Fig. 3) [47]. Also, since active FOXO1 is a negative regulator (inhibitor) of SREBP-1c expression [48], inactivation of FOXO1 would elevate SREBP-1c expression causing increased hepatic triglyceride synthesis. Collectively, these mechanisms favour hepatic lipid accumulation (Fig. 3).

Betaine reduces the “extent of demethylation of protein phosphatase-2A catalytic C subunit” [45], thereby attempting to rescue its methylation level. This would help restore FOXO1 activity and prevent hepatic lipid accumulation (Fig. 3) [45].

An additional mechanism of relevance is the alcohol-induced increment in cellular ceramide, which increases the activity of protein phosphatase-2A, which in turn inhibits AMPK activity (inhibits AMPK phosphorylation) and reduces AMPK levels. This contributes to hepatic steatosis [40]. Restoration of AMPK activity by betaine [34] would reduce de novo lipogenesis, restore β-oxidation of fatty acids, and thus attenuate hepatic steatosis (Fig. 3).

Apparently, the two aforementioned observations: alcohol-induced increment in the activity of protein phosphatase-2A [40] and alcohol-induced reduction in methylation of protein phosphatase-2A [45] may seem contradictory. This is because formation of functional protein phosphatase-2A involves its methylation by leucine carboxyl methyltransferase-1 [49]. This implies that reduction in protein phosphatase-2A methylation would reduce its activity. The reason for these seemingly contrasting inferences remains to be investigated and explained. Regardless of the reason, existence of both events has been shown and these events contribute to hepatic steatosis (Fig. 3).

Table 1 provides a brief overview of betaine dosing in various clinical trials and experiments in animal models and cell lines.

**Table 1:** Overview of betaine dosing in some clinical trials and experiments in animal models and cell lines

Betaine dosage in various experiments/trials	Reference
Exposure of betaine from diet is approximately 0.830 g/day. Exposure of 4 g/day for 6 months showed no adverse effects on platelet counts in human. Exposure of 0.4 g/day betaine in addition to the endogenous exposure is considered safe for human [50]	
Clinical trials	
Oral, 1,3,6 g single doses mixed with orange juice after overnight fast	[51]
Oral, 3 g/day for 1 month	[9]
Oral, 10 g, twice a day, probably for 12 months	[13]
Oral, 20 g/day for 12 months	[52]
3–9 g/day for different conditions for a mean of 7.4 ± 4.3 years	[16]
Oral, betaine glucoronate in combination with diethanolamine glucuronate and nicotinamide ascorbate for 8 weeks	[53]
Oral, 20 g/day in 2 divided doses for 12 months	[54]
Oral, 10 g (anhydrous betaine) twice a day for 12 months	[55]
Animal experiments	
Piglets: 20 g/kg feed (2% betaine in diet) for 6 weeks	[50]
Rats: ethanol with 1% betaine for 6 months	[7]
Rats: 10 and 50 mg/kg i.p for three consecutive days	[17]
Rats: ethanol containing water with betaine (1% w/v) for 6 months	[18]
Rats: betaine 0.4 g/kg/day intragastrically post 12 weeks of high-fat diet from weeks 13–16	[19]
Rats: ethanol with betaine (1% w/v; 10 mg/mL) for 4–5 weeks	[23]
Rats: ethanol with 1% betaine for 4 weeks	[28]
Rats: ethanol with 1% betaine for 4 weeks	[43]
Rats: 3 g/kg betaine hydrochloride in diet for up to 14 days	[44]
Rats: 1% betaine either from 2 weeks before or after partial hepatectomy	[14]
Rats: 1% betaine w/v in water for 3 weeks	[27]
Guinea pigs: Betaine containing chow (2% w/w) for 30 days	[21]
Mice: 0.5% betaine w/v in liquid diet for 5 weeks	[31]
Mice: 1% w/v betaine in drinking water for 16 weeks	[34]
Mice: 2% betaine/100 g diet for 7 weeks	[36]
Cell lines	
Betaine at 2 mM (with or without fatty acids) to HepG2 cells and AML12 cells for 24 h	[24]
Betaine at 2 mM (with or without alcohol) to HepG2 cells overexpressing CYP2E1 for 24 h	[56]
Betaine at 84 mM to 336 mM to Hepa 1–6 and clones of HepG2 cells for up to 4 days	[57]
Betaine up to 80 mM to breast cancer cell line MCF-7, in combination or parallel with ethanol treatment for 6 days	[58]
Rat small intestinal cell line IEC-18 3.4 to 6.8 mM for 24 h (in combination with lipopolysaccharide)	[59]

## Summary and future work

Betaine promises attenuation of alcohol-induced hepatic steatosis in humans. It acts as a lipotrope by increasing mitochondrial fatty acid oxidation, restoring VLDL synthesis and secretion, and reducing de novo lipogenesis as well as hepatic uptake of adipose-derived free fatty acids. Essentially, by enhancing and restoring hepatic methylation potentiation, it regulates genes/proteins that prevent triglyceride accumulation in the liver. Since majority of contextual studies have been conducted in animal models and in cell culture, elaborate clinical trials are needed to affirm the results and examine whether the beneficial effects of betaine vary with age, sex, and ethnicity.

It is extremely surprising that despite the therapeutic potential of betaine, there are insufficient contextual studies in humans. For example, as of August 2021, PubMed shows only a couple of published randomized control trials that have shown promising effects of betaine in non-alcoholic fatty liver disease [52, 53] and there are a couple of clinical trials for non-alcoholic steatohepatitis [54, 55]. There is a desperate and urgent need for inexpensive and non-toxic therapeutic agents that can pause the exacerbation of alcohol-induced hepatic steatosis. The reason for lack of sufficient relevant studies in human remains unknown.

## Data Availability

Not applicable.
